# COMPLICATIONS AND LATE FOLLOW-UP OF SCOPINARO’S SURGERY WITH GASTRIC PRESERVATION: 1570 PATIENTS OPERATED IN 20 YEARS

**DOI:** 10.1590/0102-672020210002e1646

**Published:** 2022-06-17

**Authors:** Paula VOLPE, Carlos Eduardo DOMENE, André Valente SANTANA, William Giglio MIRA, Marco Aurélio SANTO

**Affiliations:** 1 Integrated Center for Advanced Medicine, Surgery - CIMA - Sao Paulo, São Paulo, Brazil;; 2 Hospital de Clinicas, Faculty of Medicine, University of Sao Paulo, São Paulo, Brazil

**Keywords:** Postoperative Complications, Bariatric surgery, Malnutrition, Morbid obesity, Complicações Pós-Operatórias, Cirurgia Bariátrica, Desnutrição, Obesidade Mórbida

## Abstract

Scopinaro-type biliopancreatic diversion (BPD-S) and its variations are the surgeries that offer the best immediate results in weight loss and regain in the late follow-up. It has a high rate of immediate complications and demands control with frequent laboratory tests.

**AIMS:**

The aim of this study was to analyze the late postoperative complications of 1570 patients operated by biliopancreatic diversion with gastric preservation laparoscopic video with up to 20 years of postoperative follow-up.

**METHODS:**

In a follow-up period of up to 20 years, the clinical and surgical complications of 1570 patients with grade II or III obesity were evaluated who were operated on from 2001 to 2014 with the same team of surgeons. Clavien Dindo 11 classification was used for analysis and comparison. Laboratory tests and body mass index (BMI) were used in the analysis of late metabolic outcomes.

**RESULTS:**

On the one hand, complications in 204 patients were recorded (13%), and 143 patients (9.1%) were reoperated. On the other hand, 61 patients (29.9%), who had postoperative complications were clinically treated with good evolution in 9.2 years (95%CI 8.2-10.3), with a median of 9.5 years (95%CI 6.1-12.9). Gastroileal anastomosis ulcers occurred in 44 patients (2.8%). Patients with malnutrition, severe anemia, or chronic diarrhea were operated on with common loop elongation (n=64 - 4%), conversion to gastric diversion (n=29 - 5%), or reversal of surgery (n=10 - 0.6%). One death was registered throughout casuistry (0.06%).

**CONCLUSIONS:**

Metabolic result of DBP-S was considered excellent in most patients, even referring to changes in the frequency of bowel movements, loose stools, and unpleasant odor. Complications are usually serious and most of the patients require surgical treatment. Therefore, the biliopancreatic diversion of Scopinaro should be reserved for exceptional cases, as there are safer surgical alternatives with less serious side effects.

## RESUMO - RACIONAL -

A derivação biliopancreática tipo Scopinaro (DBP-S) e suas variações são as cirurgias que oferecem os melhores resultados imediatos na perda de peso e de reganho de peso no acompanhamento tardio. Apresenta índice elevado de complicações imediatas e demanda controle laboratorial frequente.

**OBJETIVOS -:**

Analisar as complicações pós-operatórias tardias de 1.570 pacientes operados por derivação biliopancreática com preservação gástrica videolaparoscópica com até 20 anos de acompanhamento pós-operatório.

**MÉTODOS -:**

Foram avaliadas as complicações clínicas e cirúrgicas, no acompanhamento tardio de até 20 anos, de 1.570 pacientes com obesidade grau II ou III, operados no período de 2001 a 2014, por uma mesma equipe de cirurgiões. A classificação de Clavien Dindo 11 foi empregada para análise e comparação. Exames laboratoriais e índice de massa corpórea (IMC) foram utilizados na análise dos resultados metabólicos tardios.

**RESULTADOS -:**

Foram registradas complicações em 204 pacientes (13%), e 143 (9,1%) foram reoperados. Por outro lado, 61 pacientes (29,9%), no período de 9,2 anos (IC 95% 8,2-10,3), com mediana de 9,5 anos (IC 95% 6,1-12,9), que tiveram complicações pós-operatórias foram tratados clinicamente, com boa evolução. A úlcera de anastomose gastroileal ocorreu em 44 pacientes (2,8%). Os pacientes com desnutrição, anemia grave ou diarreia crônica foram operados com alongamento da alça comum (n=64 - 4%), conversão para *bypass* gástrico (n=29 - 5%) ou reversão da cirurgia (n=10 - 0,6%). Foi registrado um óbito ao longo de toda a casuística (0,06%).

**CONCLUSÕES -:**

O resultado metabólico da DBP-S foi considerado excelente na maioria dos pacientes, mesmo referindo-se a alteração da frequência de evacuações, fezes amolecidas e com odor desagradável. As complicações são geralmente graves e a maioria demanda tratamento cirúrgico. Portanto, a derivação biliopancreática de Scopinaro deve ser reservada a casos excepcionais, pois existem alternativas cirúrgicas mais seguras e com efeitos colaterais menos graves.

## INTRODUCTION

Currently, bariatric surgeries are performed to determine different outcomes in terms of weight loss and maintenance. Scopinaro-type biliopancreatic diversion (BPD-S), the biliopancreatic diversion with duodenal deviation (DBP-DD), and its variations are the surgeries that achieve the best immediate results in weight loss and the lowest rate of weight regain in the late follow-up; in addition, they also determine the best remission rates and prolonged control of type 2 diabetes mellitus (DMII) and dyslipidemia. On the contrary, they are more complex surgeries and more difficult to be performed, have a greater rate of immediate complications, and also require frequent pathological testing due to a significant decrease in vitamins and minerals, in addition to the increased risk of protein malnutrition. Quality of life is compromised by flatulence, diarrhea, and foul odor in feces mainly due to steatorrhea caused by lower fat absorption. Such complications continue to occur even after more than 20 years of follow-up ^4^ ^,^ ^12^ ^,^ ^35^ in different moments of the postoperative period, which apparently are not predictable; these patients are reoperated for clinical complications or reviews for malnutrition or poor quality of life due to diarrhea and flatulence ^28^.

The above-mentioned factors, technical complexity, and a high rate of complications partially help in explaining the low adherence of surgeons to biliopancreatic leads, which never exceeded 2% of all bariatric procedures performed worldwide ^2^ ^,^ ^5^ ^,^ ^6^.

### Objectives

The present work analyzes postoperative complications and delayed results from 1570 patients operated by biliopancreatic diversion with gastric preservation laparoscopic video (Domene et al., 2001) with up to 20 years of postoperative follow-up.

## METHODS

### Casuistic

**Figure 1 -:**
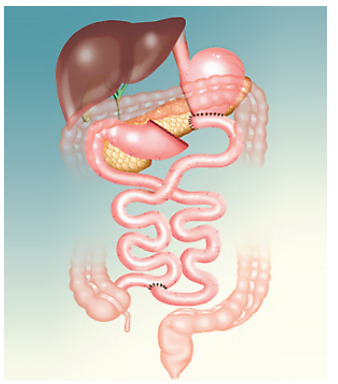
Biliopancreatic diversion with gastric preservation.

A total of 1570 patients with grade II or III obesity were retrospectively evaluated. These patients were operated in the period from 2001 to 2014, whose data were collected from medical records. All patients have undergone biliopancreatic diversion with gastric preservation laparoscopic video, with gastric reservoir of 200-400 ml, food loop of length 150-200 cm, and common loop of length 100-120 cm, according to previously published standardization ^13^ (Figure 1).

**Figure 2 -:**
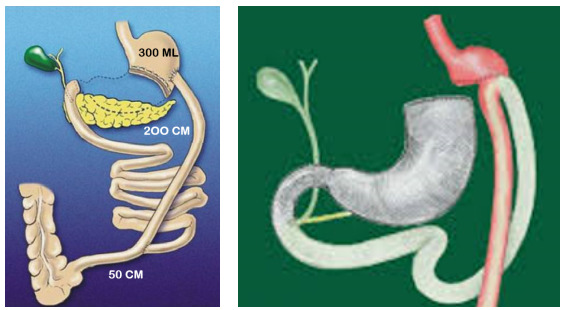
Biliopancreatic diversion surgery (BPD-S) described by Scopinaro et al. (1979) ^33^ on the left and BPD-S described by Mason and Ito (1967) ^25^ on the right.

This surgery is a modification of the biliopancreatic diversion proposed by Scopinaro et al. (1979) ^30^, who performed distal gastrectomy having a common loop of 50 cm in length; this surgery was based on the proposal by Mason and Ito (1967) who performed gastroenterostomy having a loop of 25 cm (Figure 2).

Of all the patients, 1366 (87.0%) had no complications, while 204 (13.0%) developed postoperative complications; 61 patients (29.9%) were clinically treated and 143 (70.1%) had undergone surgical treatment. These complications will be correlated with the time of onset of treatment. There was a second complication in 36 of these patients (17.6%).

The data analysis process of this research began with an exploration descriptive that resulted in frequency tables for qualitative variables. Statistical descriptive values, such as mean, standard deviation, median, and interquartile range (IIQ), were calculated in order to summarize continuous or discrete quantitative variables. Kolmogorov-Smirnov test was used to assess the probability distribution of the quantitative attributes. When necessary, quantitative variables were categorized according to the expansion of the analytical possibilities. Clavien Dindo 11 classification was used in the analysis of complications.

To test the hypothesis of non-modification of quantitative attributes related to control of diabetes mellitus between groups with a significant diagnosis of DM, a repeated-measure ANOVA model was used ^21^.

Kaplan-Meier curves were used to estimate the probability of occurrence of complications after treatment. All tests considered one to bidirectional 0.05 and a 95% confidence interval (CI) and were performed using the computational software R (https://www.r-project.org/) package nparLD, IBM SPSS ^25^ (Statistical Package for the Social Sciences), and Excel 2016 ^®^ (Microsoft Office) ^27^.

The study in question received approval from the Ethics Committee under the number 31002620.9.0000.0068 at the Hospital de Clinicas of the Faculty of Medicine of the University from Sao Paulo.

## RESULTS

There were complications in 204 patients (13%), and some patients had more than one complication (Table 1).

**Table 1 -:** Postoperative complications of 204 patients (there were patients with more than one complication).

Complication	Number	%
Malnutrition	95	46.5
Chronic Diarrhea	58	28.4
Gastroileal Anastomosis Ulcer	44	21.5
Severe Chronic Anemia	42	20.5
Internal Hernia	21	10.3
Acute Pancreatitis	03	0.14
Arthritis	02	0.90
Hepatopaty	02	0.90
Spontaneous Bone Fracture	02	0.90
Pulmonary Tuberculosis	02	0.90
Intestinal Tuberculosis	01	0.50
Idiopathic Septicemia	01	0.50
Intractable Hipocalcemia	01	0.50
Intestinal Obstruction	01	0.50
Total	288	

Tables 2 and 3 summarize the characteristics of patients who evolved with complications. These individuals were mostly female (145 - 71.1%) (95%CI 64.6-77.0), with a mean age of 40.0 years (±13.0 years). According to Table 2, 57 or (27.9%) (95%CI 22.1-34.4) of the individuals were diabetic at T0 interval; 143 cases were affected by surgical complications (70.1%) (95%CI 63.6-76.1), also characterized as Clavien Dindo IIIB. After the treatment of the first complication, 36 individuals (17.6%) developed a second complication, classified as Clavien Dindo IIIB in 19 cases (52.8%; 95%CI 36.8-68.3).

**Table 2 -:** Characteristics of individuals with complications, including absolute and relative frequency and 95% confidence interval (95%CI)

	N	%	95%CI	
			Inferior	Superior
Sex				
Male	59	28.9%	23.0%	35.4%
Female	145	71.1%	64.6%	77.0%
Diabetes T0				
No	147	72.1%	65.6%	77.9%
Yes	57	27.9%	22.1%	34.4%
Complication Type T1				
Clinical	61	29.9%	23.9%	36.4%
Surgical	143	70.1%	63.6%	76.1%
Clavien Dindo T1				
II	61	29.9%	23.9%	36.4%
IIIB	143	70.1%	63.6%	76.1%
Treatment T1				
Clinical	61	29.9%	23.9%	36.4%
Surgical	143	70.1%	63.6%	76.1%
Evolution T1				
Good	188	92.2%	87.9%	95.3%
Regular	14	6.9%	4.0%	10.9%
Death	2	1.0%	0.2%	3.1%
Complications T2				
I	1	2.8%	0.3%	12.3%
II	14	38.9%	24.3%	55.2%
IIIB	19	52.8%	36.8%	68.3%
IV	2	5.6%	1.2%	16.6%
Treatment T2				
Clinical	12	42.9%	27.6%	59.3%
Surgical	24	57.1%	40.7%	72.4%
Evolution T2				
Good	34	94.3%	82.9%	98.8%
Death	2	5.7%	1.2%	17.1%

**Figure 3 -:**
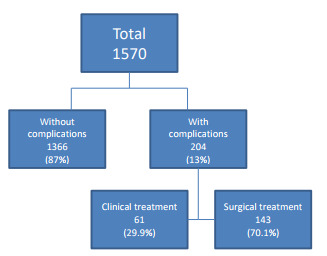
Occurrence of complications among operated patients.

BMI measurements in the group with complications (CC) ranged from 41.2 kg/m^2^ (±13.0 kg/m^2^) at T0 to 27.9 kg/m^2^ (±4.8 kg/m^2^) at T3, while hemoglobin measurements, ferritin, and albumin were 10.8 g/dL (±1.8 g/dL), 110.3 μg /L (±228.3 μg /L), 3.3 g/dL (±0.9 g/dL) in T1 to 10.3 g/dL (±1.7), 197.6 μg /L (±449.7), and 3.2 g/dL (±0.8 g/dL), respectively (Table 3).

**Table 3 -:** Descriptive statistics of individuals with complications (CC) considered in the study, such as mean, standard deviation (SD), median, 25th (P25) and 75 (P75) percentiles, minimum (Min.), and maximum (Max.)

	Average	SD	Median	P25	P75	Min.	Max.
Age (years)	48.0	10.0	48.0	42.0	54.0	17.0	72.0
Time of disease (years)	4.7	3.3	4.0	2.0	6.0	0.4	20.0
BMI (kg/m^2^) T0	42.0	5.8	40.8	37.7	44.7	30.1	65.2
A1C (%) T0	7.9	1.5	7.3	6.8	8.4	6.5	14.8
Glycemia (mg/dL) T0	161.0	58.0	140.0	124.0	183.0	102.0	576.0
Other medications (number) T0	1.0	1.0	1.0	1.0	2.0	1.0	4.0
Comorbidities (number) T0	2.0	1.0	1.0	1.0	2.0	1.0	6.0
A1C (%)T1	5.6	0.9	5.5	5.2	6.0	4.0	10.3
Glycemia (mg/dL) T1	96.8	22.9	93.0	87.0	101.5	4.9	189.0
A1C (%) T2	5.0	0.6	5.0	4.6	5.4	3.6	7.2
Glycemia (mg/dL) T2	92.2	16.2	88.0	84.0	96.0	70.0	193.0
BMI (kg/m^2^) ST	30.7	5.2	29.7	27.5	33.2	19.7	57.1
A1C (%) ST	5.3	0.9	5.2	4.6	5.7	3.5	10.0
Glycemia (mg/dL) ST	95.0	25.7	89.5	85.0	99.0	48.0	317.0
Other medications (number) ST	0.0	1.0	0.0	0.0	0.0	0.0	3.0
Comorbidities ST	1.0	0.0	1.0	1.0	1.0	1.0	2.0

Gastroileal anastomosis ulcer occurred in 44 patients (21.5%), 34 of them without complications and 23 of them with complications such as perforation, stenosis, or upper gastrointestinal bleeding (Table 4).

**Table 4 -:** Complications of gastroileal anastomosis ulcers.

Gastroileal anastomosis ulceration	N	%
No perforation, bleeding, or stenosis	17	08.3
Perforation	17	08.3
High digestive bleeding	07	04.9
Stenosis	03	01.4
Total	44	21.5

The percentages refer to the total number of patients with complications (n=204).

Notably, 143 (70.1%) patients had undergone surgical treatment. There was death in 0.49% of patients. The surgeries performed in 143 patients who had complications in surgical procedures are listed in Table 5. Clinical treatment was indicated for 61 (29.9%) of the patients who had postoperative complications, all with good evolution after the treatment was performed (Table 6).

**Table 5 -:** Management of 143 patients with surgical complications (diversion is an abbreviation for Roux-en-Y gastric bypass).

Diagnosis	N	%	Treatment	N	%	Evolution
Malnutrition/Anemia	68	33.3	Common Limb Elongation	55	26.9	Good
			Conversion To Bypass	10	04.9	Good
			Reversal	03	01.5	Good
Chronic Diarrhea	32	15.6	Conversion To Bypass	16	07.8	Good
			Common Limb Elongation	09	04.4	Good
			Reversal	07	03.4	Good
Internal Hernia	21	10.3	Mesenteric Closure	21	10.3	Good
GI Anastomosis Ulcer	17	08.3	Suture Of Ulceration	07	03.4	Good
			Degastrectomy	07	03.4	Good
			Conversion To Bypass	03	01.5	Good
Intestinal Obstruction	01	0.49	Enterectomy	01	0.49	Good
Acute Pancreatitis	01	0.49	Distal Pancreatectomy	01	0.49	Death
Spontaneous Fracture	01	0.49	Reversal	01	0.49	Good
Acute Hepatopathy	01	0.49	Reversal	01	0.49	Good
Hipocalcemy	01	0.49	Reversal	01	0.49	Good
Total				143	70.1	

**Table 6 -:** Complications that were treated clinically (the percentage refers to the total number of patients with complications, n=204)

Diagnosis	N	%	Evolution
Severe malnutrition	28	13.7	Good
Gastroileal anastomosis ulceration	27	13.2	Good
Severe chronic diarrhea	02	0.98	Good
Septicemy	01	0.49	Good
Spontaneous bone fracture, malnutrition, anemia	01	0.49	Good
Pulmonary tuberculosis, malnutrition, ulcer	01	0.49	Good
Acute hepatic failure	01	0.49	Good
Total	61	29.9	

**Figure 4 -:**
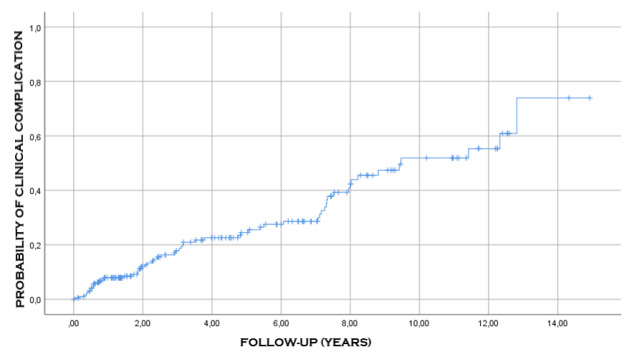
Probability of occurrence of clinical complications after the surgical procedure.

Among 204 individuals who suffered a complication, with 61 cases accounting for 29.9% of the total complications, the average time of occurrence of clinical complications was 9.2 years (95%CI 8.2-10.3), with a median of 9.5 years (95%CI 6.1-12.9); the incidence of these complications was proportional to time, that is, at 3 years of follow-up, the probability of occurrence of this event was 20%, and at 8 years of follow-up, the probability was 40% (Table 7 and Figure 4).

**Table 7 -:** Time before the occurrence of post-surgery complications, including absolute frequency, relative mean, and median estimates with 95% confidence interval (95%CI)

	N	%	Average	95%CI	Median	95%CI
Clinical complication	59	29.9%	9.2	8.2-10.3	9.5	6.1-12.9
Surgical complication	140	70.1%	5.9	5.2-6.6	5.1	3.7-6.5
Ulcer	51	21.5%	10.1	9.1-11.2	11.7	8.9-14.5
Malnutrition	89	43.6%	7.6	6.8-8.5	7.5	6.3-8.8
Internal hernia	22	10.3%	12.4	11.5-13.3	14.3	9.8-18.8

Malnutrition was observed just after the treatment (T0), as it occurred in an average of 7.6 years (95%CI 6.8-8.5). Ulcers were observed in an average of 10.1 years (95%CI 9.1-11.2) and internal hernia occurred in an average of 12.4 years after surgery (95%CI 11.5-13.3).

**Figure 5 -:**
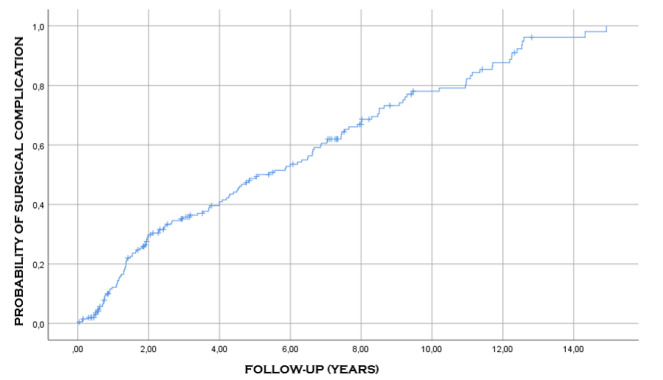
Probability of occurrence of surgical complications after the surgical procedure.

Complications that required reoperation after surgery, that is, those classified as Clavien-Dindo IIIB, with 143 cases, accounted for 70.1% of cases of post-treatment complications. The average time of occurrence of complications until the presence of this event was 5.9 years (95%CI 5.2-6.6), with a median of 5.1 years (95%CI 3.7-6.5). Approximately 35% of cases with complications occurred up to the second year after surgery, with proportionality observed in the time after the second year, which extended to the tenth year. Even after this period, some cases of reoperation occurred (Figure 5).

**Figure 6 -:**
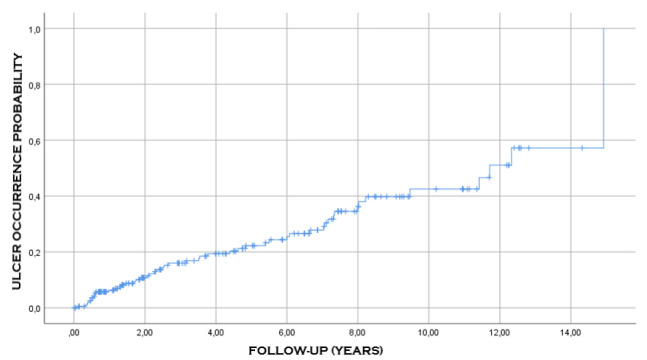
Probability of occurrence of ulcer after the surgical procedure.

**Figure 7 -:**
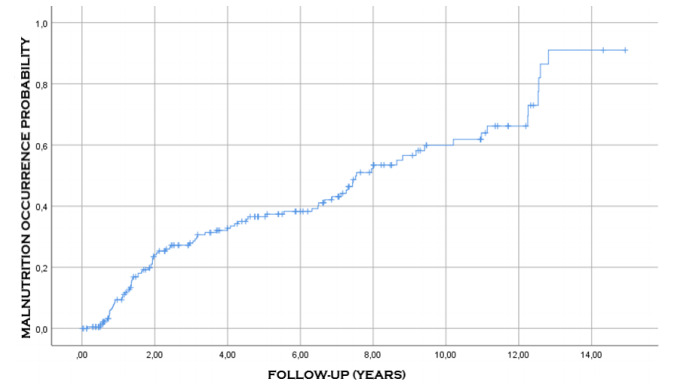
Probability of occurrence of malnutrition after the surgical procedure.

**Figure 8 -:**
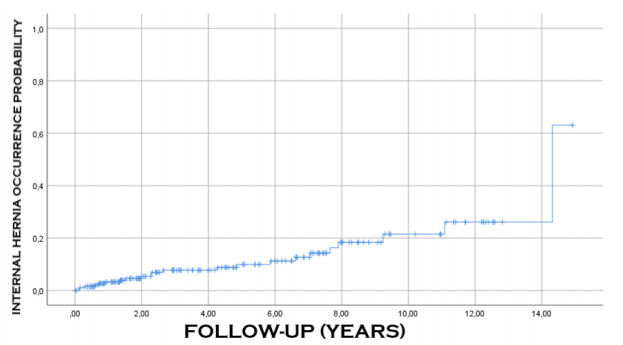
Probability of occurrence of internal hernia after the surgical procedure.

Figures 6, 7 and 8 show the probability of occurrence of ulcer, malnutrition, and internal hernia after surgery, respectively. It can be noted that these events occurred consecutively in 44 (21.5%), 89 (43.6%), and 21 (10.3%) of the patients, respectively.

Thirty-six patients had a second complication (17.6%). The diagnosis of the first complication in these patients is summarized in Table 8.

**Table 8 -:** Diagnosis of the first complication in patients with the second complication.

First complication	N	%		N	%
Malnutrition	19	09.3	Associated	11	05.3
			Isolated	08	03.9
GI anastomosis ulcer	11	05.3	Perforation	06	02.9
			No perforation	05	02.4
Diarrea				03	01.4
Internal hernia				03	01.4
Total				36	17.6

Among 36 patients who had a second complication, three had severe chronic diarrhea and three had internal hernias as the first complication. The behavior at first complication, and diagnosis, management, and evolution of the second complication are summarized in Table 9.

**Table 9 -:** Diagnosis of the first complication and respective management; diagnosis, management, and evolution of second complication in patients whose first complication was diarrhea or internal hernia.

First complication	N	Treatment	Second complication	N	Treatment	N	Evolution
Diarrhea	3	Common Limb Elongation	Diarrhea	3	Conversion to bypass	3	Good
Internal Hernia	2	Mesenteric Closure	Gastroileal anastomosis ulcer	2	Clinical	2	Good
Internal Hernia	1	Mesenteric Closure	Malnutrition	1	Reversion	1	Good

Of the 19 patients who had malnutrition as their first complication, five were treated clinically and 14 underwent surgery on the first occasion. Diagnosis, treatment, and evolution of the second complication in patients with malnutrition and clinical treatment are summarized in Table 10.

**Table 10 -:** Diagnosis and management in the second complication of patients whose first complication was malnutrition (n=19) and had clinical treatment (n=5).

Second complication	N	Treatment	Evolution
Gastroileal anastomosis ulcer	2	Clinical	Good
Desnutrição	2	Clinical	Good
Internal hernia	1	Enterectomy and mesenteric closure	Good
Total	5		

Exceptionally, 14 malnourished patients who underwent surgery were treated with stretching of the common loop through the section of the anastomosis of the alimentary loop at the level of the anastomosis with the ileum, and anastomosis of the alimentary loop at 1.5 m from the biliopancreatic loop, counted from the broken anastomosis (Figure 8).

The second complication, management, and evolution of these patients are summarized in Table 11.

**Table 11 -:** Diagnosis and management of the second complication of patients whose first complication was malnutrition (n=19), and they were treated with stretching the loop (n=14)

Second complication	N	Treatment	N	Evolution
Perforated ulcer	3	Reversal	1	Good
		Degastrectomy	1	Good
		Ulcer suture	1	Good
Ulcer without perforation	2	Clinical	2	Good
Malnutrition	3	Clinical	2	Good
		Reversal	1	Good
Diarrhea	2	Conversion to bypass	2	Good
Internal hernia	1	Mesenteric closure	1	Good
Ulcerative colitis	1	Colectomy and reversal	1	Good
Hepatic failure	1	Clinical	1	Death
Hepatic cirrosis	1	Hepatic transplantation	1	Death
Total	14		14	

Eleven patients had gastroileal anastomosis ulceration as the first complication, six of them with perforation and five of them without perforation. The evolution and conduct of second complication of these patients are summarized in Tables 12 and 13.

**Table 12 -:** Evolution of the five patients who had the ulcer of gastroileal anastomosis without perforation as their first complication.

Treatment of First complication	N	Second complication	Treatment	Evolution
Clinical	1	Upper GI bleeding	Hemostasis	Good
Clinical	1	Malnutrition	Clinical	Good
Clinical	1	Internal hernia	Mesenteric Closure	Good
Degastrectomia	1	Anastomosis ulcer	Clinical	Good
Degastrectomia	1	Malnutrition	Common limb elongation	Good
Total	5			

**Table 13 -:** Evolution of the six patients who had the gastroileal anastomosis ulcer with perforation as their first complication.

Treatment of First complication	N	Second complication	Treatment	Evolution
Clinical	1	Upper GI bleeding	Hemostasis	Good
Clinical	1	Malnutrition	Clinical	Good
Clinical	1	Internal hernia	Mesenteric Closure	Good
Degastrectomy	1	Anastomosis ulcer	Clinical	Good
Degastrectomy	1	Malnutrition	Common limb elongation	Good
Total	5			

## DISCUSSION

In an effort to reduce the serious side effects of pure intestinal diversions for the treatment of morbid obesity, Scopinaro et al. (1979) ^33^ modified the procedure of gastric diversion proposed by Mason et al.; Ito (1967) ^25^ performed a horizontal subtotal gastrectomy, one gastroileal and one ileoileal anastomosis (Figure 2).

Compared to the study by Mason et al., the surgery performed in the study by Ito uses a larger gastric reservoir, long biliopancreatic loop, and small common loop, associating less restriction to food intake, the relative decrease in absorption of carbohydrates, and a large malabsorption of proteins and fats. After the experimental studies were carried out in animals, the authors standardized a technique in humans with a gastric reservoir of 200-500 ml, loop feeding from 200 to 300 cm, and a common handle of 50 cm ^33^.

The biliopancreatic diversions are the operations that promote more and more sustained weight loss in the late follow-up, as well as effective and prolonged control of the DMII 14.32. In the present study, the result in terms of weight loss was very satisfactory, starting from an average of 42.0 kg/m^2^ in the preoperative period to 30.7 kg/m^2^ in the last consultation after surgery.

The mean glycated hemoglobin was 7.9 g/dl preoperatively and 5.0 g/dl in the late follow-up (Table 3). However, the rate of late complications was 13%, with malnutrition being the most common, followed by diarrhea, gastroileal anastomosis ulcer, and anemia; the inpatient care for hernia also occurred in 21 patients during late follow-up (Table 1). The majority of these complications were very severe, classified as Clavien-Dindo IIIB in 70.1% of cases (Table 2). Often, the increase in the size of the common loop did not determine the resolution of the complication - whether malnutrition, diarrhea, or anemia - which requires a new reintervention.

Gastrojejunal anastomosis ulcers occur between 3.2% and 12.5% of patients after BPD-S; the production of hydrochloric acid in the large gastric stump is a characteristic of this surgery, which is observed in the pathogenesis of ulcer. A decrease in the number of duodenal ulcers in BPD-S can be explained by the absence of acid exposure to the duodenum; a possible explanation would be the obstruction of the afferent loop and consequent ischemia ^16^. The complications resulting from BPD-S, such as malnutrition (16%), anastomotic ulcers (16%), and reversal of surgery (8%), have no different incidence when patients are classified according to the difference in age ^8^.

Gastroileal anastomosis ulcers were observed in 2.8% of cases in our study (Table 4). Active follow-up of these patients was carried out, with the performance of endoscopies noticed at 6 months, 12 months, and annually thereafter. It should be noted that the high incidence of anastomotic ulcer in BPD-S was the main reason for the description of biliopancreatic diversion with duodenal deviation (DBP-DD) ^24^. Also, more than half of our patients had ulcer complications, such as perforation, hemorrhage, or stenosis, often leading to the need for reoperation for its treatment.

Surgical modifications in the digestive tract to control morbid obesity promote a decrease in the absorption surface of the intestine, creating conditions for a state of malabsorption ^32^. This can be increased in some cases, manifesting itself as clinically significant micronutrient or macronutrient deficiencies. Serious calorie-protein deficiencies, requiring nutritional support, are observed in about 5% of patients with gastric diversion (BPG), increasing from 20% to 30% in patients with BPD, reflecting the important malabsorption induced by these procedures ^34^. The handle feed and the short common handle represent a smaller surface for absorbing nutrients and consequently pose a potential risk of protein, vitamin, and mineral deficiencies; as glucose is well absorbed in all the intestinal segments, there is no risk of lack of glucose. Vitamin and mineral deficiencies after BPD-S and BPD-DD are a big problem: up to 90% of these patients will develop some type of vitamin or mineral deficiency within 3 years after surgery ^19^. This relates to the characteristic of large desorption of these surgeries due to the varying modalities of the common channel of 50-100 cm ^20^. It is essential to consider the long length of the small intestine outside the food transit represented by the biliopancreatic loop. In this handle, there can be bacterial overgrowth, leading to several consequences^26^.

Symptoms include diarrhea and weight loss, which can be erroneously attributed to the change in anatomical effect of the gastrointestinal tract caused by the surgery, and such complication may be underdiagnosed ^17^. Garzon et al. (2007) ^17^ demonstrated that intestinal loop lengths determine important differences in terms of weight loss and complications. The authors compared two groups of patients operated on for BPD-S with loop intestinal measurements: a group with a length of 50 cm of common loop and a length 200 cm of food loop and another group with a length of 75 cm of common loop and a length of 225 cm of food loop were followed up for 12 years. The first group had better and more sustained weight loss; however, this same group presented much more malnutrition (16%) and anemia (60%) than the second group (2% and 40%, respectively).

Several modifications of Scopinaro’s surgery have been published, to decrease the rate of morbidity and late complications. In one of these modifications, bowel loops were having similar length, along with the preservation of the distal stomach, to perform a less aggressive intervention and reduce morbidity ^3^ ^,^ ^9^ ^,^ ^13^ ^,^ ^29^. The modification by Domene et al. (2001) ^13^ was used in the present study. This procedure includes a gastric reservoir of 200-400 ml, length of food loop of 200 cm, and length of common loop from 50 to 100 cm, without resection of the distal stomach, aiming to reduce surgical trauma and avoiding the risk of fistula of the duodenal stump. Consecrated surgeries, such as the Roux-en-Y gastric diversion, or gastric diversion, preserving the distal stomach, showed the safety of these types of surgeries. The type of gastric preservation performed in our patients does not pose a risk of retained antrum syndrome, as it preserves the acid inside the stomach, due to the small size of the gastric stump. The DBP-S determines a high incidence of gastroileal anastomosis ulcer, and this occurred in 44 patients in our sample (2.8%), many of them difficult to treat and even needing surgical treatment (Table 1).

Crea et al. (2011)^9^ compared 287 patients operated on with BPD-S with distal gastrectomy and 253 with gastric preservation and length of 50 cm of common loop, for more than 7 years. The two groups had similar results in terms of weight loss and resolution of diabetes; according to the authors, there were no vitamin and protein deficiencies in this follow-up period. There were 13 cases (2.4%) of anastomotic ulcers in this group, six with gastrectomy and seven without resection, with no statistically significant difference.

In the study by Ballesteros-Pomar et al. (2016), ^3^ 299 patients underwent surgery, 71 (24%) with distal gastrectomy and 228 (76%) with gastric preservation, with length of food loop around 200 cm and length of common loop ranging from 50 to 100 cm, followed for 10 years. The length of common loop was initially 50 cm and was later increased to 100 cm to reduce nutritional complications. No significant differences were found between clinical and nutritional complications among patients with or without gastrectomy, as well as length of common loops of 50 or 100 cm in extension. After 10 years, the loss of excess weight was 63.7%; blood glucose levels and cholesterol were normal in all the patients. Protein malnutrition affected 4% of patients and anemia occurred in 16% of patients during the follow-up period; 61.5% of patients had vitamin deficiency during the follow-up. Vitamin A, D, and E deficiencies were increased in the late follow-up. There was no study on the occurrence of ulcers of anastomosis.

Follow-up of 75 patients operated without distal gastrectomy showed that the results obtained in 11 patients were quite similar to those of Scopinaro - anemia in 78.6% of cases, hypoproteinemia in 25.4% of cases, and hypovitaminosis in less than 10% of patients. Clinical occurrences such as diarrhea, flatulence, and anal diseases were also frequent ^23^ ^,^ ^30^.

Another serious consequence of biliopancreatic diversion is liver cirrhosis, which has the absorption of hepatotoxic substances in the excluded small intestine as one of its mechanisms, in the context of bacterial overgrowth, protein malnutrition, and excessive mobilization of free fatty acids, causing steatosis and oxidative damage of the hepatocytes ^22^, which may even lead to liver transplantation ^10^.

Of the complications that occurred in 13% of our patients, 61 of the patients (29.9%) had undergone clinical treatment, and 143 (70.1%) were treated surgically (Tables 4-6). Our patients were instructed preoperatively and postoperatively as per the need for the use of intense and continuous supplementation of vitamins and minerals. Even so, the iron deficiency and fat-soluble vitamins were very common, often requiring the use of injectable replacement, mainly iron. Except for patients with anastomotic ulcers or septicemia, who had hospitalization of up to 2 weeks, all other clinically treated patients had a prolonged period of hospitalization, from 4 weeks to 4 months, for nutritional and clinical recovery. Enteral nutrition does not show good results in these patients, as it leads to severe diarrhea even with elemental nutrition; all of these patients needed prolonged parenteral nutrition for their recovery. There was good evolution in all of them, but some developed a second complication during late follow-up (Table 8).

Among the patients who had undergone surgical treatment (Table 5), all those who had a hernia were treated with the closure of the mesenteric gap and had a good evolution. Seventeen patients with perforated ulcers were treated, with seven of them treated with only ulcer raffia, seven treated with degastrectomy, and three with conversion to gastric diversion. Patients with malnutrition, severe anemia, or chronic diarrhea were operated on with elongation of the common loop (n=64), conversion to gastric diversion (n=29), or reversal of surgery (n=10). The reversal was always carried out at the request of the patients. Initially, it was the elongation of the common loop which was performed in all patients; some of them did not have satisfactory evolution and required reintervention, leading to the indication of conversion to gastric diversion, all with good results. The only operated patient who died was one with severe acute pancreatitis which progressed to hemorrhagic necrosis.

**Figure 9 -:**
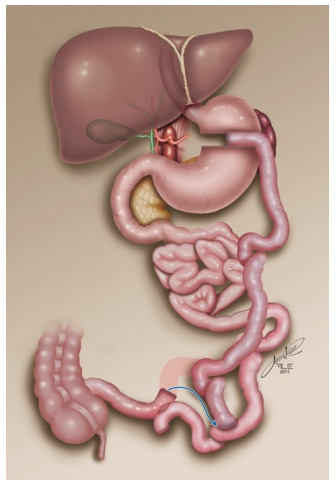
Illustration of the elongation of the common loop through the jejunoileal anastomosis section, anastomosing the alimentary loop 1.5 m above the biliopancreatic loop.

The elongation of the common loop was performed by sectioning the loop anastomosis feeding at the level of the anastomosis with the ileum, and anastomosis of the alimentary loop at 1.5 m of the biliopancreatic loop, counted from the broken anastomosis (Figure 9). Thereby, the absorption surface is increased by incorporating a 1.5 m biliopancreatic loop in the common loop, through which the digested food passes.

Complications, both clinical and surgical, occur in about one-third of the cases (35%) in the first 2 years, but they continue to happen even after 15 years of surgery. The mean time of surgical complications was 5.9 years, and that of clinical complications was 9.2 years (Table 7, Figures 4 and 5). Malnutrition, anastomotic ulcers, and internal hernias can happen even after 15 years of surgery (Figures 6-8). These observations demonstrate the need for permanent monitoring of these patients, as they can present serious complications even after a long time after surgery. A second complication occurred in 36 patients who had malnutrition, anemia, diarrhea, or internal hernia as their first complication (Table 9); 12 of them were treated clinically and 24 were treated surgically (Tables 9-13).

The second complication was mostly different from the first one. Three patients with the closure of the breach due to internal hernia evolved with anastomotic ulcer or malnutrition. Three of them with diarrhea and elongation of the common loop during the first complication continued having diarrhea and were converted to diversion (Table 9). Among 19 patients with malnutrition as the first occurrence, 5 had undergone clinical treatment initially and 14 were treated with common loop elongation (Tables 10 and 11). Of those who had clinical treatment initially, two of them again had malnutrition and were clinically managed; other two patients had ulcers of anastomosis and one with an internal hernia was treated surgically (Table 10).

Of the 14 patients operated with loop stretching, 5 had nutritional complications - malnutrition or diarrhea: three were treated with reversal or conversion to diversion, and two with parenteral nutrition. It is important to highlight the occurrence of two cases of severe liver changes that resulted in the death of these patients (Table 11). Even with the significant increase in the area of ​​nutrient absorption that occurred again with severe nutritional complications or liver alterations resulting from the modification of the enterohepatic bile salts, these occurrences motivated the conversion to diversion as an option of choice in patients with severe nutritional complications or diarrhea.

Among 11 patients diagnosed with an anastomotic ulcer in the first complication and had a second complication, 5 of them had no perforation, and 6 had a perforation in the first complication. Of the five patients without drilling, there was a new ulcer in two of them, malnutrition in the other two, and an internal hernia in one of them (Table 12). The other six patients had perforation, which initially evolved with a new ulcer in two of them, malnutrition in another two, and an internal hernia in one (Table 13). The ulcer of anastomosis continues to occur in the late postoperative period and is possibly due to performing the Roux-en-Y gastroileal anastomosis, with a relatively long-term gastric stump.

The results with the various length modifications of the intestinal loops of the Scopinaro surgery demonstrated the difficulty of striking a balance between the effects of surgery, such as sufficient and sustained weight loss, and serious side effects such as malnutrition, anemia, and multiple vitamin deficiencies; the greater the weight loss, the higher the risk of serious complications. The evaluation of the various publications with modifications of the lengths of the intestinal loops of the DBP-S, to maintain adequate slimming power with minimal side effects, did not bring very different results. These types of surgeries determine an important improvement in the metabolic syndrome -- control of blood glucose, cholesterol, and triglyceride levels - and a consistent reduction in excess weight, maintained in the late postoperative period. One should follow general, unrestricted diet, which is an important factor in assessing the quality of life by the operated patients. However, they have very worst results regarding the symptoms of side effects, nutritional effects, and micronutrient levels. The patients often present with diarrhea, foul odor of feces and skin, in addition to pathologies of orifices and anastomotic ulcers. Albumin levels and fat-soluble vitamins (A, D, E, K), in addition to calcium, iron, and zinc, are greatly altered and need continuous replacement. They need constant monitoring of changes to avoid clinical and nutritional complications. These changes can even present themselves 20 years after surgery, and these patients need reoperations to control clinical and nutritional complications ^15^. Modifications of the Scopinaro’s surgery, such as the biliopancreatic diversion with duodenal diversion (DBP-DS), also have difficulties similar to the basic Scopinaro’s surgery to establish the lengths of bowel loops with the right balance between unwanted side effects and adequate leakage and sustained weight.

No other surgery has satisfactory results to control obesity such as the one carried out by biliopancreatic diversions - DBP-S or DPB-DD. However, studies showed that the proportion of DBP-DD has been decreasing from 6.1% to 4.9%, and 2.1% in 2003, 2008, and 2011, respectively ^7^, corresponding to less than 1% of all bariatric surgeries. ^18^ Notably, 1187 (0.6%) of 215666 patients were operated in the USA in 2016 ^14^.

As the procedure that determines the best and maximum sustained weight loss, significant reversal of comorbidities, is the least performed surgery in the world? The answer is multifactorial and complex. First, it is a highly complex surgery and requires a skilled and experienced team. The morbidity and mortality of this surgery is the highest among all modalities of surgical treatment of obesity, in which mortality may reach 2.7%, against 0.1% of the most commonly performed surgeries ^1^.

Our experience with 1570 patients operated on and followed up for up to 20 years shows that the metabolic result of BPD-S is excellent in most patients; however, practically, there are significant changes in the frequency of bowel movements as the feces are passed easily but with an unpleasant odor that often forces the patient to limit their social life, having difficulty going to public restrooms due to the bad smell of feces and, also, to have a bathroom isolated from the house for their personal use. Also, the skin has a change in smell, which can be very strong, and the more intense the odor, the greater the fat intake by the patient.

The replacement of trace elements and vitamins need to be continuous and intense. Vitamin D is permanently low, and even with high-dose replacement, it hardly reaches normal values. Mild anemia happens in most patients, and all need parenteral iron replacement in late follow-up. Malnutrition and severe diarrhea almost always lead to long hospital stays and need prolonged parenteral nutrition; enteral nutrition is either insufficient or cannot be performed because it causes severe diarrhea, possibly due to lesions of the intestinal mucosa by malnutrition. Complications occur in a large number of cases; they are usually serious and most of them require surgical treatment. Due to all these complications, DBP-S should be reserved for exceptional cases, as there are safer surgical alternatives with less serious side effects.

## CONCLUSIONS

The metabolic result of DBP-S was considered excellent in most of the patients when referring to changes in the frequency of bowel movements, loose stools, and an unpleasant odor. Complications occur in a large number of severe cases and most of them require surgical treatment. Therefore, the derivation of Scopinaro’s biliopancreatic diversion should be reserved for exceptional cases, as there are safer surgical alternatives with less serious side effects.
